# A single-center clinical study on the combination of Five-flavor sophora flavescens enteric-coated capsules and vedolizumab in induction therapy for ulcerative colitis

**DOI:** 10.3389/fimmu.2026.1791086

**Published:** 2026-04-22

**Authors:** Xinglang Li, Linyan Zhou, Feng Tian, Hui Li, Ying Xie

**Affiliations:** Department of Gastroenterology, Shengjing Hospital of China Medical University, Shenyang, Liaoning, China

**Keywords:** efficacy, Five-flavor sophora flavescens enteric-coated capsules, safety, ulcerative colitis, vedolizumab

## Abstract

**Objective:**

The aim of this study was to evaluate the efficacy and safety of Vedolizumab (VDZ) combined with Five-flavor sophora flavescens enteric-coated capsules (FSEC) for induction therapy in Ulcerative colitis (UC).

**Methods:**

UC patients treated with FSEC and/or VDZ were enrolled and divided into combination group (VDZ+FSEC) and single drug group (VDZ). The primary end points were clinical and endoscopic response and remission rates at week 14 and week 52. Secondary end points were the 14-week corticosteroid withdrawal rate, the number of bowel movements, the number of bloody stools, improvement in inflammatory markers, and safety.

**Results:**

The clinical response rate (97.4% vs. 79.1%, P = 0.011, 95% confidence interval(CI) 4.1%, 32.8%) and endoscopic response rate (74.4% vs. 51.2%, P = 0.031, 95%CI 2.2%, 41.4%) at week 14 in the combination group were significantly higher than those in the single-agent group. PP analysis showed that the endoscopic response rate at week 52 in the combination group was significantly better than that in the monotherapy group (85.7% vs. 62.5%, P = 0.042, 95%CI 2.0%, 44.4%), but there was no significant difference in ITT analysis. At week 14, the number of bowel movements and hematochezia scores were significantly improved in the combination group and the monotherapy group, and the improvement in the combination group was more significant at early stage and multiple time points. There were no serious adverse reactions in the two groups.

**Conclusion:**

FSEC combined with VDZ demonstrated superior clinical response rates and endoscopic response rates, and had greater advantages in early improvement of hematochezia and defecation frequency, compared to VDZ monotherapy, with a favorable safety profile.

## Introduction

Ulcerative colitis (UC) is a chronic nonspecific inflammatory disease of the colonic mucosa, characterized by continuous mucosal inflammation extending proximally from the rectum (1, 2). Current therapeutic agents for UC are 5-Aminosalicylic acid (5-ASA) preparations, glucocorticoids, immunosuppressants, and biologic agents.

Multiple studies have confirmed the mechanism of action of traditional Chinese medicine (TCM) in regulating inflammatory responses in the treatment of UC. TCM has been used to treat UC, and the immune system plays a key role in regulating intestinal homeostasis (3). Five-flavor sophora flavescens enteric-coated capsules (FSEC) is a kind of Chinese traditional medicine, which is compound preparation comprising five herbal components: Sophora flavescens, Sanguisorba officinalis, Indigo naturalis, Bletilla striata, and Glycyrrhiza uralensis. This formulation synergistically exerts anti-inflammatory, antipruritic, and hemostatic effects through heat-clearing and dampness-drying, detoxification and ulcer healing, and cooling blood to arrest bleeding mechanisms. Preclinical studies demonstrate that FSEC suppresses Th17 cell-driven autoimmune inflammation by modulating IL-17A, IL-6, IL-1β, and TNF-α signaling pathways (4). FSEC also has been clinically proven to be effective in treating UC (5). Constructing a screening model to obtain the functional herbs for the treatment of active ulcerative colitis based on herb-compound-target network showed that Sophorae Flavescentis Radix (Kushen) was found to be capable of relieving abdominal pain and hematochezia during active UC (6).

Vedolizumab (VDZ), a gut-selective monoclonal antibody targeting α4β7 integrin, exhibits favorable long-term safety but delayed onset of action (7, 8).

In clinical practice, we observed rapid symptomatic improvement (e.g., alleviation of abdominal pain and rectal bleeding) in patients with moderate-to-severe UC treated with VDZ combined with FSEC. Currently, no relevant studies exist on the combination of FSEC with VDZ. This study analyzes and compares the clinical efficacy and safety of FSEC combined with VDZ versus VDZ alone in patients with UC, aiming to provide a theoretical basis for clinical application.

## Materials and methods

This single-center cohort study enrolled patients with moderate-to-severe ulcerative colitis (UC) treated with vedolizumab (VDZ) alone or in combination with Sophora flavescens Pentaphyllum Enteric-Coated Capsules (FSEC) at Shengjing Hospital of China Medical University between January 2021 and October 2024. The study protocol was approved by the Institutional Review Board of Shengjing Hospital of China Medical University (Ethics Approval No.: 2024PS1658K). Inclusion criteria: patients with moderate to severe active UC aged 18–75 years old; moderate to severe active UC is defined as a Mayo score>6, with rectal bleeding score>1 and Mayo endoscopic subscale>1; first use of biological agents; patients treated with VDZ are given medication at weeks 0, 2, and 6, and then every 8 weeks thereafter; patients treated with FSEC for at least 8 weeks. Exclusion criteria: patients in the inactive phase; individuals with organic lesions in the intestine, severe complications, or other anal diseases that affect drug evaluation; Individuals with diarrhea caused by infectious diarrhea, CD, ischemic colitis, radiation colitis, parasitic infections, etc.; patients with severe primary diseases such as liver, kidney, hematopoietic system, endocrine system, and psychiatric disorders; Alanine aminotransferase (ALT) and aspartate aminotransferase (AST) levels exceeding twice the upper limit of normal reference values.

The study was divided into a combination group (VDZ + FSEC) and a monotherapy group (VDZ). The combination group received VDZ 300 mg via intravenous infusion at weeks 0, 2, and 6, followed by every 8 weeks thereafter, combined with FSEC (4 capsules orally three times daily) for a minimum of 8 weeks, while the monotherapy group received VDZ 300 mg via intravenous infusion at weeks 0, 2, and 6, followed by every 8 weeks thereafter. Clinical response rates, clinical remission rates, endoscopic response rates, and endoscopic remission rates were compared between the two groups at weeks 14 and 52. Biochemical parameters, stool frequency, hematochezia improvement, and adverse events were recorded. For patients missing week 52 colonoscopy assessments, both Per-Protocol Set (PP) analysis and Intention-To-Treat (ITT) analysis were employed to calculate clinical response rates, clinical remission rates, endoscopic response rates, and endoscopic remission rates. Steroid withdrawal rates at week 14 were compared between the two groups among patients who underwent steroid induction therapy.

The study employed the following definitions:

1. Clinical response was defined as a reduction of ≥30% or ≥3 points in the modified Mayo score from baseline, accompanied by a ≥1-point decrease in the rectal bleeding sub-score or an absolute rectal bleeding sub-score of 0 or 1; the clinical response rate was calculated as the percentage of patients achieving clinical response relative to the total population.
2. Clinical remission was defined as a modified Mayo score ≤2 points with no individual sub-score >1 point, and the clinical remission rate was calculated as the percentage of patients achieving clinical remission relative to the total population.
3. Endoscopic response was defined as a reduction of ≥1 point in the Mayo endoscopic sub-score, with the endoscopic response rate calculated as the percentage of patients achieving endoscopic response relative to the total population.
4. Endoscopic remission was defined as a Mayo endoscopic sub-score ≤1 point, and the endoscopic remission rate was calculated as the percentage of patients achieving endoscopic remission relative to the total population.
5. The week 14 steroid withdrawal rate was defined as the percentage of patients who discontinued steroid therapy by week 14 among those who received steroid induction treatment.

### Statistical analysis

Statistical analysis was performed using SPSS 27.0 software. Normally or approximately normally distributed measurement data were expressed as mean ± standard deviation (X̄ ± S), with between-group comparisons conducted using t-tests; non-normally distributed measurement data were presented as medians and analyzed with rank-sum tests for intergroup comparisons; categorical data were described using frequencies and percentages, with Chi-square tests employed for between-group comparisons. A threshold of P<0.05 was considered statistically significant.

## Results

### Baseline characteristics

A total of 82 UC patients were enrolled in this study, comprising 39 patients (46.1%) in the combination group and 43 patients (53.9%) in the monotherapy group, with their baseline clinical and demographic characteristics detailed in **Table 1**.

**Table 1:** Baseline characteristics of enrolled patients.

Parameter	Combination group (n=39)	Monotherapy group (n=43)	P-value
Male, n(%)	19(47.0%)	24(55.8%)	0.521
Age, years	46.9±16.7	51.8±15.7	0.107
Body weight, kg	61.2±12.6	60.0(49.3,68.8)	0.691
BMI	21.9±3.5	21.4(18.5,26.3)	0.817
Smoking history			0.235
Yes, n(%)	3(7.7%)	7(16.3%)	
No, n(%)	36(92.3%)	36(83.7%)	
Disease extent (Satsangi et al., 2006)			0.436
E1, n(%)	0	0	
E2, n(%)	8(20.5%)	12(27.9%)	
E3, n(%)	31(79.5%)	31(72.1%)	
Disease severity			0.330
Moderate, n(%)	25(64.1%)	23(53.5%)	
Severe, n(%)	14(35.9%)	20(46.5%)	
Corticosteroid use	16(41.0%)	17(39.5%)	0.891
CRP(mg/L)	3.9(1.5, 7.4)	3.7(2.0, 9.5)	0.616

**Figure 1:**
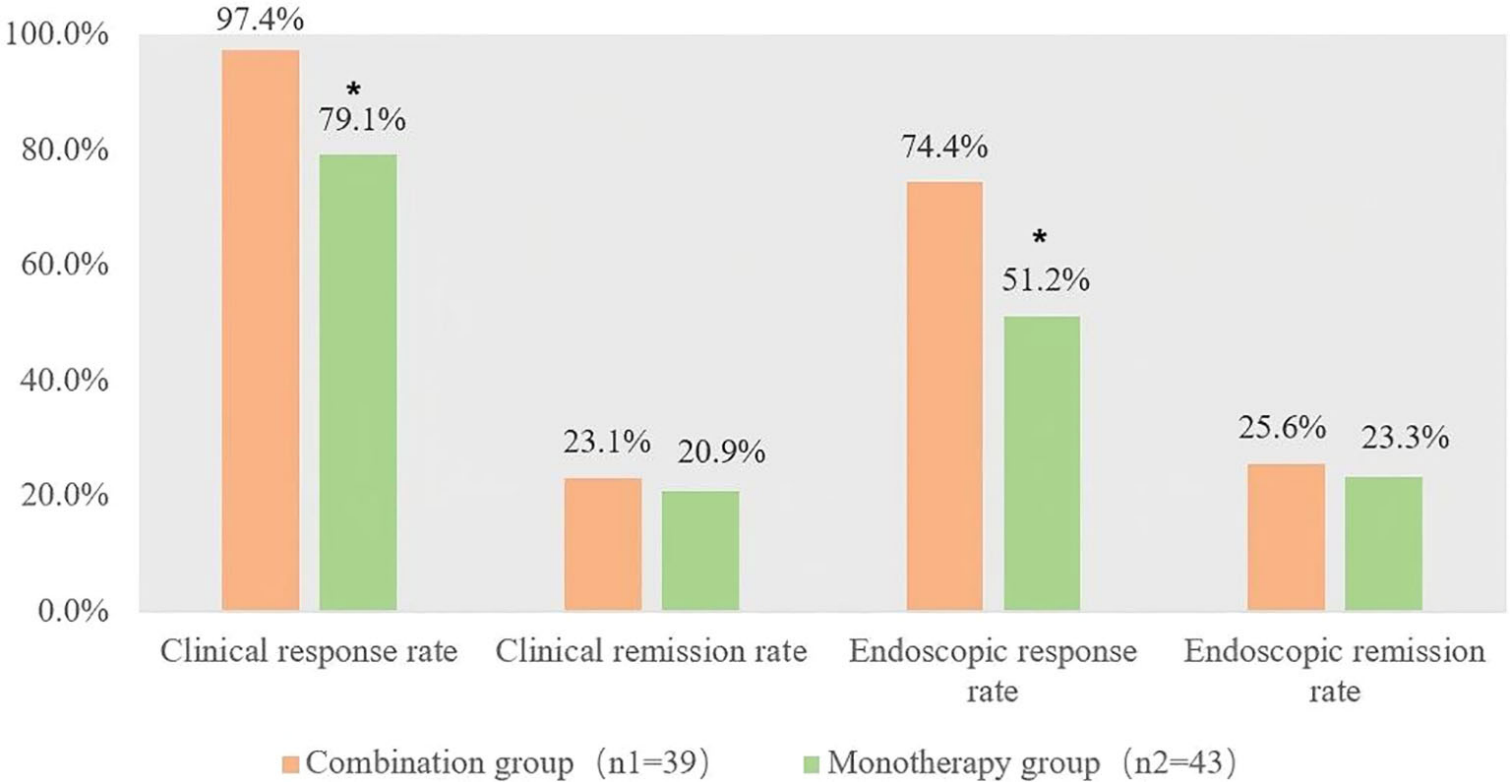
Clinical efficacy assessment at week 14. *p<0.05, compared with the combination group.

At week 14, 38 patients (97.4%) in the combination group achieved clinical response, compared to 34 patients (79.1%) in the monotherapy group, demonstrating a statistically significant difference (P = 0.011, 95% confidence interval(CI) 4.1%, 32.8%); similarly, 29 patients (74.4%) in the combination group attained endoscopic response versus 22 patients (51.2%) in the monotherapy group, with a statistically significant intergroup difference (P = 0.031, 95%CI 2.2%, 41.4%); however, clinical remission rate and endoscopic remission rate showed no statistically significant difference between two groups (**Figure 1**).

Per-Protocol (PP) analysis demonstrated that 24 patients (85.7%) in the combination group achieved endoscopic response at week 52, compared to 20 patients (62.5%) in the monotherapy group, with a statistically significant difference (P = 0.042, 95%CI 2.0%, 44.4%) (**Table 2**).

**Table 2:** Clinical efficacy at week 52 (Per-Protocol analysis).

Time		Combination group (n=28)	Monotherapy group (n=32)	χ² Value	*P*
Week 52	Clinical response rate	100.0%(28)	93.8%(30)	1.810	0.178
	Clinical remission rate	50.0%(14)	40.6%(13)	0.530	0.466
	Endoscopic response rate	85.7%(24)	62.5%(20)	4.115	0.042
	Endoscopic remission rate	57.1%(16)	43.8%(14)	1.071	0.301

In the intention-to-treat (ITT) analysis at week 52, 24 patients (61.5%) in the combination therapy group achieved endoscopic response, compared with 20 patients (46.5%) in the monotherapy group, demonstrating no statistically significant difference between the groups (P = 0.173, 95%CI -6.3%, 36.3%) (**Table 3**).

**Table 3:** Clinical efficacy at week 52 (Intention-to-Treat analysis).

Time		Combination group (n=39)	Monotherapy group (n=43)	χ² Value	*P*
Week 52	Clinical response rate	71.8%(28)	69.8%(30)	0.041	0.840
	Clinical remission rate	35.9%(14)	30.2%(13)	0.297	0.586
	Endoscopic response rate	61.5%(24)	46.5%(20)	1.857	0.173
	Endoscopic remission rate	41.0%(16)	32.6%(14)	0.632	0.427

For patients who, due to personal reasons or changes in their condition, did not strictly undergo colonoscopy at the 52-week time point, we used the Last Observation Carried Forward (LOCF) method to impute missing values for those missing 52-week colonoscopy data. Specifically, the colonoscopy results closest to week 52 were used to fill in the missing value for each subject, assuming that the disease status remained stable during the missing period. Although missing data were imputed, the differences still did not reach statistical significance (**Table 4**).

**Table 4:** Clinical efficacy at week 52 (LOCF).

Time		Combination group (n=39)	Monotherapy group (n=43)	χ² Value	*P*
Week 52	Clinical response rate	100.0%(39)	100%(43)	0	1
	Clinical remission rate	59.0%(23)	51.2%(22)	0.504	0.478
	Endoscopic response rate	82.1%(32)	69.8%(30)	1.674	0.196
	Endoscopic remission rate	56.4%(22)	48.8%(21)	0.470	0.493

**Figure 2:**
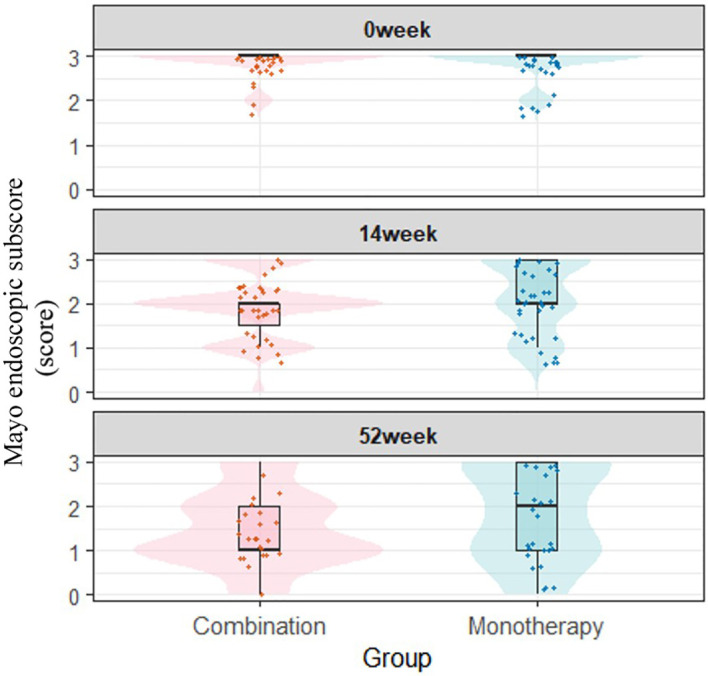
Longitudinal comparison of Mayo endoscopic subscores between treatment groups.

**Figure 3:**
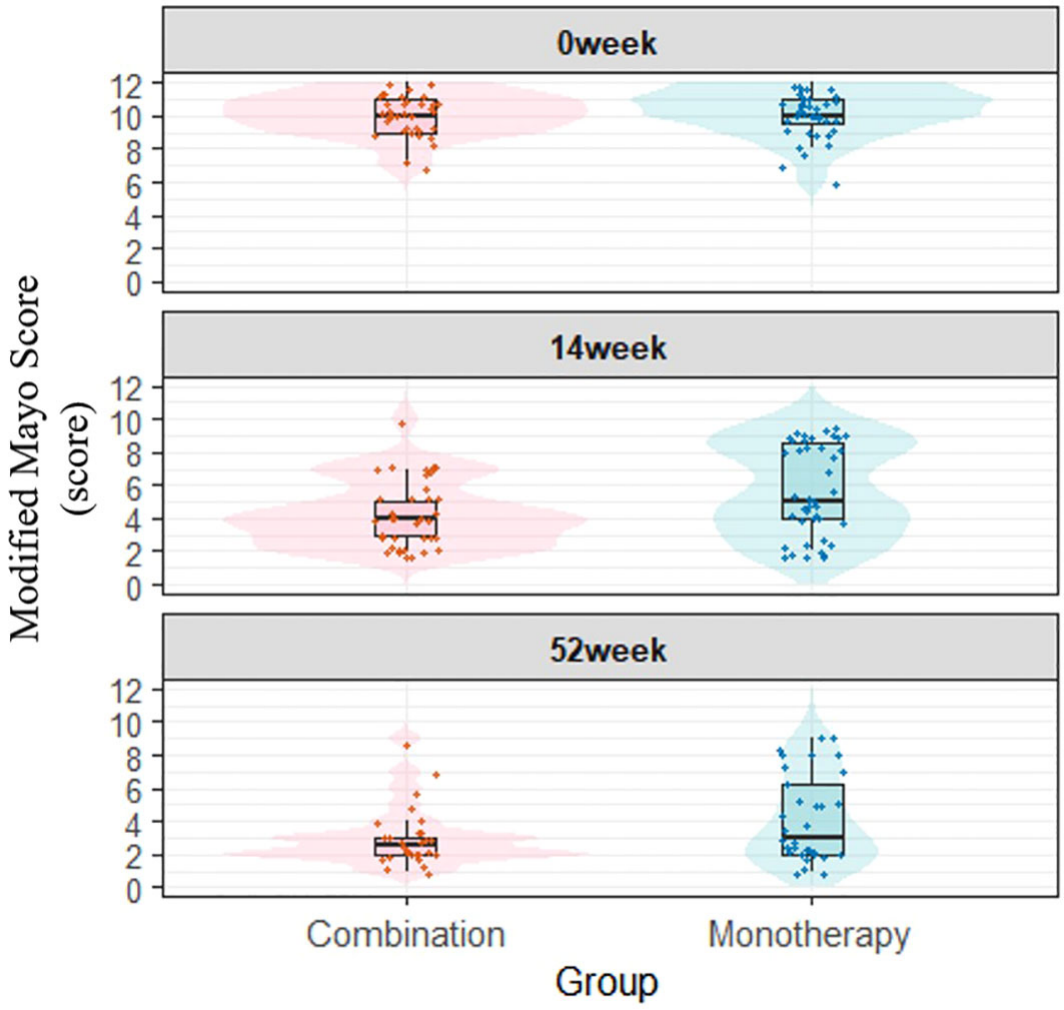
Temporal changes in modified Mayo clinical scores across study arms (Box-and-Whisker Plots).

Despite the absence of statistically significant differences in clinical efficacy between the combination therapy and monotherapy groups, visual inspection of the box plots revealed superior improvements in Mayo endoscopic subscores and modified Mayo clinical scores within the combination therapy group. (**Figures 2**, **3**).

At week 14, steroid-free remission was achieved in 12 patients (75.0%, 12/16) in the combination therapy group and 11 patients (64.7%, 11/17) in the monotherapy group, with no statistically significant difference observed between groups (χ²=0.414, P = 0.520) (**Table 5**).

**Table 5:** Steroid-free remission rate at Week 14.

Time		Combination group (n=16)	Monotherapy group (n=17)	χ² Value	*P*
Week 14	Steroid-free remission rate	75.0%(14)	64.7%(11)	0.414	0.520

Both intergroup and intragroup comparisons revealed statistically significant differences in the stool frequency subscore and rectal bleeding subscore of the modified Mayo score at weeks 2, 6, and 14 between the combination therapy and monotherapy groups (**Tables 6**, **7**).

**Table 6:** Intergroup comparisons of stool frequency and rectal bleeding subscores in the modified mayo score before and after treatment.

Assessment time point	Combination group (n=39)	Monotherapy group (n=43)	*Z*	*P*
Stool Frequency Subscore Baseline (0 week)	2(2, 3)	2(2, 3)	-0.841	0.400
Stool Frequency Subscore Week 2	1(0, 1)	1(0, 1)	-2.681	0.007
Stool Frequency Subscore Week 6	0(0, 0)	0(0, 1)	-3.789	<0.001
Stool Frequency Subscore Week 14	0(0, 0)	0(0, 1)	-4.259	<0.001
Rectal Bleeding Subscore Baseline (0 week)	3(2, 3)	2(2, 3)	-1.298	0.194
Rectal Bleeding Subscore Week 2	1(1, 1)	1(1, 2)	-4.545	<0.001
Rectal Bleeding Subscore Week 6	0(0, 1)	1(0, 1)	-2.764	0.006
Rectal Bleeding Subscore Week 14	0(0, 1)	0(0, 2)	-2.207	0.027

**Table 7:** Intra-group comparison of modified mayo score subscores for stool frequency and rectal bleeding before and after treatment.

Group		Stool frequency subscore week 2 vs baseline	Stool frequency subscore week 6 vs baseline	Stool frequency subscore week 14 vs baseline	Rectal bleeding subscore week 2 vs baseline	Rectal bleeding subscore week 6 vs baseline	Rectal bleeding subscore week 14 vs baseline
Combination Group (n=39)	*Z*	-6.229	-6.401	-6.286	-6.495	-6.275	-6.316
	*P*	<0.001	<0.001	<0.001	<0.001	<0.001	<0.001
Monotherapy Group (n=43)	*Z*	-6.707	-6.750	-6.413	-6.711	-6.587	-6.586
	*P*	<0.001	<0.001	<0.001	<0.001	<0.001	<0.001

During the induction treatment phase, liver function abnormalities (defined as ALT/AST elevations >3×ULN) occurred in 3 patients (7.7%) in the combination therapy group and 2 patients (4.7%) in the monotherapy group. Comparative analysis revealed no statistically significant intergroup differences in the incidence of elevated AST (χ²=1.27, P = 0.260) or ALT (χ²=0.33, P = 0.565). All transaminase elevations resolved spontaneously or with dose adjustment, with no cases progressing to severe drug-induced liver injury (DILI) or treatment discontinuation (**Table 8**).

**Table 8:** Incidence of hepatic enzyme abnormalities during induction therapy.

Parameter	Combination group (n=39)	Monotherapy group (n=43)	*X*	*P*
AST	7.7%(3)	2.3%(1)	1.269	0.260
ALT	7.7%(3)	4.7%(2)	0.330	0.565

## Discussion

Our study results showed that the combination of FSEC and VDZ demonstrated a significantly higher clinical response rate compared with VDZ monotherapy at week 14 of treatment initiation, with statistically significant differences. Although VDZ has shown good efficacy in early clinical response in UC, the addition of FSEC improved the early clinical remission rate, but showed no statistically significant differences. Due to the large amount of missing colonoscopy samples at week 52, ITT analysis would greatly reduce the positive rate of the results, so PP analysis was used for discussion. In the week 52 PP analysis, both groups almost completely achieved clinical response. In terms of clinical remission, there was no statistically significant difference, but the clinical remission rates in the combination group were higher than those in the monotherapy group at both week 14 and week 52 (23.1% vs. 20.9%; PP analysis: 50.0% vs. 40.6%). The GEMINI 1 study reported that in the induction trial, the clinical remission rate was 16.9% at week 6, and in the maintenance trial, the clinical remission rates at week 52 were 41.8% in the VDZ every 8 weeks group and 44.8% in the VDZ every 4 weeks group (9). The clinical remission rate of our monotherapy group was similar to the results of the GEMINI 1 trial, confirming the reliability of our observations to a certain extent.

The combination group demonstrated greater superiority in early clinical response than in clinical remission compared with the monotherapy group, reflecting that FSEC may have certain advantages in improving clinical symptoms in the early stage of treatment. We analyzed hematochezia and stool frequency during the induction phase, and results showed that the combination group had significantly better improvement in the single-item hematochezia score than the monotherapy group at weeks 2, 6, and 14, with the most pronounced effect at week 2 (all differences statistically significant: P<0.001, P = 0.006, P = 0.027). The combination group also showed superior efficacy in single-item stool frequency scores compared with the monotherapy group at weeks 2, 6, and 14, with statistically significant differences (P = 0.007, P<0.001, P<0.001), confirming that FSEC exceled in early improvement of defecation frequency and hematochezia symptoms.

In the per-protocol (PP) analysis, the combination group exhibited higher endoscopic response rates than the monotherapy group at both week 14 and week 52 (74.4% vs. 51.2%, 85.7% vs. 62.5%), with statistically significant differences between groups (P = 0.031, P = 0.042). For endoscopic remission, while no statistically significant difference was observed, the combination group had higher remission rates than the monotherapy group in PP analysis at weeks 14 and 52 (25.6% vs. 23.3%, 57.1% vs. 43.8%). A study comparing vedolizumab (VDZ) and adalimumab in adults with moderate-to-severe UC assigned patients to 300 mg VDZ infusions every 8 weeks and reported a 39.7% rate of endoscopic improvement at week 52 (10). Another study evaluating the efficacy and safety of subcutaneous vs. intravenous VDZ observed a 52-week endoscopic improvement rate of 53.7% (11). The endoscopic remission rates in our study fell between these two reported values.

Due to missing endoscopic data among patients at week 52, we performed an intent-to-treat (ITT) analysis for clinical efficacy at week 52 to ensure the authenticity and validity of results. The analysis showed that the statistically significant endoscopic response rate observed in the per-protocol (PP) analysis at week 52 (P = 0.042) became non-significant in the ITT analysis (P = 0.173), which may be related to the high rate of endoscopic data loss and the relatively small total sample size included in the study. We used the LOCF method to impute missing values for those missing 52-week colonoscopy data. The advantage of this method is its simplicity and the preservation of sample size; however, it may underestimate true changes, and the bias may be either conservative or aggressive depending on the direction of the outcome. Although missing data were imputed, the differences still did not reach statistical significance.

Although no statistically significant differences were observed in clinical remission or endoscopic remission rates between the combination and monotherapy groups at week 14, and only the PP analysis of endoscopic response rate showed statistical significance at week 52, the combination group consistently demonstrated better clinical efficacy than the monotherapy group across all outcomes. Thus, we supplemented boxplots and violin plots of Mayo endoscopic sub-scores and modified Mayo scores at weeks 0, 14, and 52 for both groups. As visually shown in **Figures 2**, **3**, the combination group exhibited superior improvements in clinical efficacy compared with the monotherapy group.

Our research has showed during the induction treatment phase, liver function abnormalities occurred in 3 patients in the combination therapy group and 2 patients in the monotherapy group. Comparative analysis revealed no statistically significant intergroup differences in the incidence of elevated AST or ALT. In the safety aspect, the elevation of abnormal liver function values in the combination group cannot be excluded as being associated with the use of FSEC. One component of FSEC is indigo naturalis (Qingdai), whose active ingredients are indirubin and indigo. Toxicity studies have demonstrated that indirubin exhibits certain hepatotoxicity: under toxic doses of indirubin, hepatocytes show swelling, lysis necrosis, and atrophic degeneration, and in swollen hepatocytes, more pronounced swelling is associated with reduced or even absent ribonucleic acid (RNA) content (12). With the widespread clinical use of indigo naturalis, an increasing number of clinically reported cases of liver injury have been documented (13–15). Therefore, combination therapy should be used with caution in patients with preexisting liver function abnormalities or liver diseases and that close monitoring of liver function is advised.

There are certain limitations in our study. Firstly, as a retrospective study, the results have inherent limitations. Secondly, the sample size was insufficient, which limited statistical power. Although the combination group showed an apparent trend toward superiority over the monotherapy groups, not all differences reached statistical significance. Thirdly, when analyzing the clinical efficacy at week 52, we used PP analysis data for comparison, which may introduce some bias. Furthermore, many studies have confirmed the immunomodulatory effect of FSEC. Our research has showed that the combination therapy of FSEC and VDZ can effectively improve the clinical response rate. But this study did not conduct in-depth research on its mechanism of action. In the future, we can detect cytokines (such as IL17A, IL6, etc.) in the peripheral blood and intestinal mucosa of patients, further explore the mechanism of action of combination therapy, and provide more theoretical basis for combination therapy. Finally, because our study was single-center and did not include hospitals from different regions or types, our patient population has certain limitations. Future large-scale, multicenter, prospective clinical studies are needed to further demonstrate the efficacy and safety of combination therapy with VDZ and FSEC.

## Conclusion

Our study showed that compared with VDZ monotherapy, FSEC combined with VDZ improved clinical response rates and endoscopic response rates, and had greater advantages in early improvement of hematochezia and defecation frequency. No serious adverse events were observed, but caution is needed for the occurrence of abnormal liver function.

## Data Availability

The original contributions presented in the study are included in the article/supplementary material. Further inquiries can be directed to the corresponding author.
